# Liver-specific propanoate metabolism-derived 2-ethylhexanol as a novel biomarker for precise diagnosis and prognosis of hepatocellular carcinoma

**DOI:** 10.1186/s40364-025-00842-7

**Published:** 2025-10-21

**Authors:** Dongyong Lee, Jinhee Mun, Jihyun Lee, Chan Jang, Geumjong Song, Jonghyuk Yun, Kwangseock Kim, Taewan Kim, Seob Jeon, Jinmyoung Joo

**Affiliations:** 1 Department of Biomedical Engineering, Ulsan National Institute of Science and Technology, Ulsan, 44919 Republic of Korea; 2 Center for Research and Development, Oncocross Ltd, Seoul, 04168 Republic of Korea; 3 Department of Computer Science and Engineering, Ulsan National Institute of Science and Technology, Ulsan, 44919 Republic of Korea; 4 Department of Surgery, Division of Gastrointestinal Surgery, College of Medicine, Soonchunhyang University Cheonan Hospital, Cheonan, 31151 Republic of Korea; 5 Future Innovation Medical Research Center, Soonchunhyang University Cheonan Hospital, Cheonan, 31151 Republic of Korea; 6 Department of Gynecology, College of Medicine, Soonchunhyang University Cheonan Hospital, Cheonan, 31151 Republic of Korea; 7 Graduate School of Health Science and Technology, Ulsan National Institute of Science and Technology, Ulsan, 44919 Republic of Korea; 8 Center for Genomic Integrity, Institute for Basic Science, Ulsan, 44919 Republic of Korea

## Abstract

Alterations in propanoate metabolism are increasingly recognized as significant biomarkers for various liver disorders, notably hepatocellular carcinoma (HCC). To enhance diagnostic specificity for HCC, we conducted integrated binary omics and targeted metabolomics analyses, identifying liver-specific propanoate metabolism (LPM) gene signatures and associated metabolites. Leveraging data from 40 tissues and 81 cell subclasses, hepatocytes showed the highest metabolic specificity. Clinical validation using the TCGA-LIHC cohort highlighted LPM genes, notably *ALDH2* and *ABAT*, as robust prognostic markers significantly associated with poor survival outcomes. Serum metabolomic profiling of 273 patients demonstrated that elevated toluene and xylene isomers and significantly reduced 2-ethylhexanol distinctly characterize HCC, distinguishing it from liver cirrhosis, hepatitis, and other cancers. These findings emphasize the potential of LPM-based metabolic profiling as a precise diagnostic and prognostic tool, with potential implications in targeted therapeutic development for HCC.

**Supplementary Information:**

The online version contains supplementary material available at 10.1186/s40364-025-00842-7.

To the editor

Propanoate metabolism, a key pathway linking fatty acid β-oxidation and amino acid catabolism, plays essential roles in energy homeostasis and cellular detoxification under normal physiological conditions. Specifically, propionyl-CoA derived from odd-chain fatty acids and certain amino acids (valine, isoleucine, methionine, threonine) undergoes sequential conversion through methylmalonyl-CoA to succinyl-CoA, which subsequently enters the tricarboxylic acid (TCA) cycle to support ATP production and redox balance [1]. Dysregulation of this pathway can result in accumulation of toxic intermediates, mitochondrial dysfunction, and aberrant lipid peroxidation, which are increasingly recognized as contributors to liver disease progression. Indeed, alterations in propanoate metabolism are increasingly recognized for their association with various liver disorders, including hepatitis B virus (HBV), hepatitis C virus (HCV), liver cirrhosis (LC), and hepatocellular carcinoma (HCC), with HCC patients exhibiting significantly altered levels of metabolites compared to those with LC or viral hepatitis [2]. Although propanoate metabolism is also present in other tissues such as the pancreas, colon, and kidneys [3, 4], its functional impact in these sites is relatively restricte (e.g., microbial fermentation–derived short-chain fatty acids in colon, proximal tubular energy metabolism in kidney). In contrast, the liver exhibits unique enrichment and regulatory specificity in this pathway, positioning hepatocytes as the central hub for systemic propanoate flux. This distinction emphasizes the necessity of investigating liver-specific propanoate metabolism (LPM) as a basis for identifying diagnostic and prognostic biomarkers uniquely indicative of liver pathology.

Herein, we performed an integrated analysis aimed at identifying LPM gene signatures and assessed their potential prognostic significance in HCC progression. Utilizing a comprehensive binary omics network, we analyzed the expression profiles of propanoate metabolism-related genes across 40 distinct human tissue types and 81 specific cell subclasses (Fig. 1A). Our analyses clearly indicated that hepatocytes exhibit the highest propanoate metabolism specificity among all examined cell subclasses. Moreover, liver tissue demonstrated markedly elevated tissue-specific expression, accounting for 45.5% (15/33 genes) of the total identified propanoate metabolism-related genes (Fig. 1B).

**Fig. 1:**
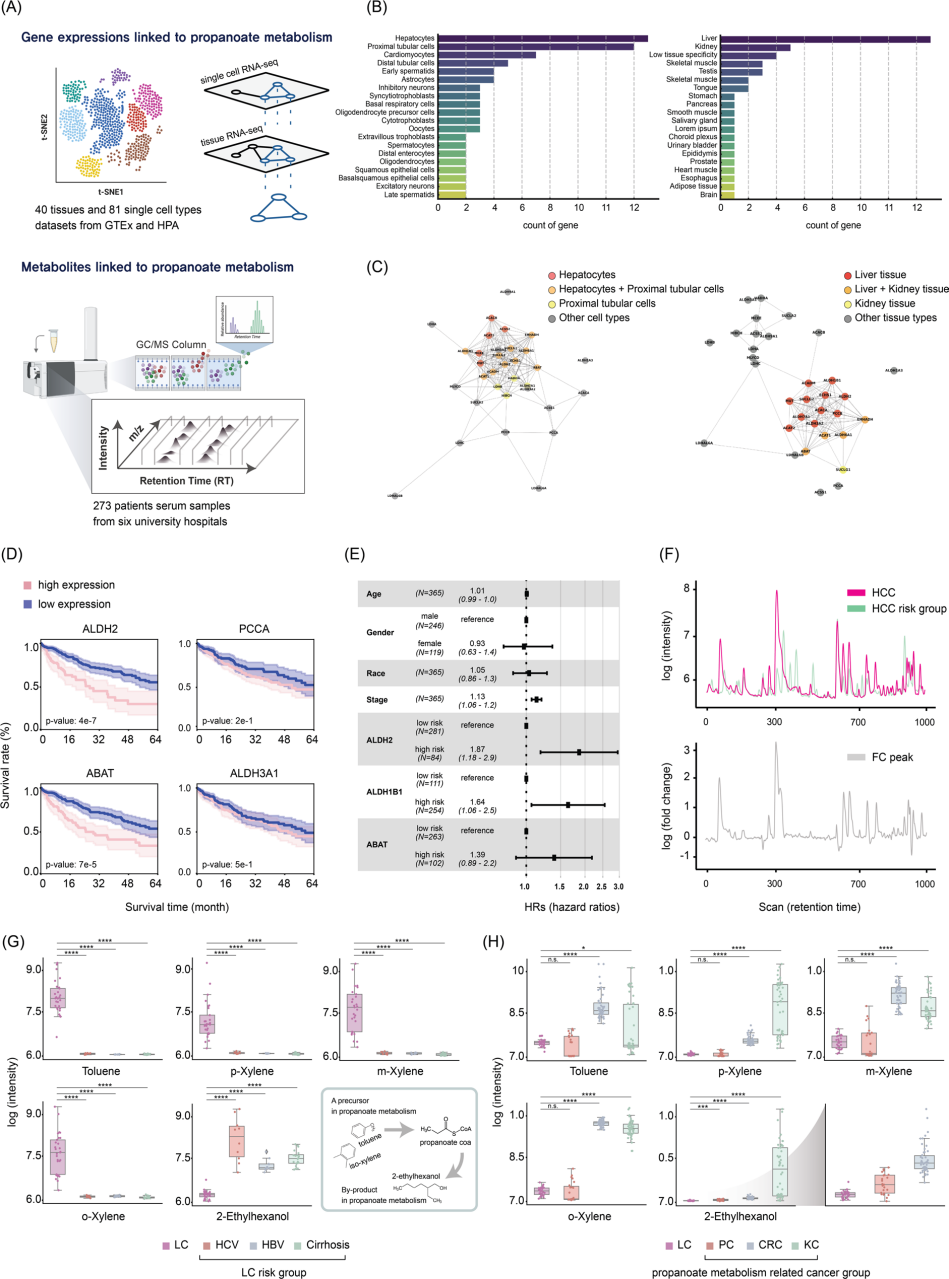
Analysis of propanoate metabolism-related gene expression and metabolite levels in liver diseases and cancers. (**A**) Schematic representation of (i) the identification process for liver-specific gene signatures within the propanoate metabolism pathway, utilizing a binary omics network approach that integrates multi-modal data to identify tissue-specific expression patterns, and (ii) the metabolomic profiling of patient serum samples for metabolic biomarker identification and diagnosis. (**B**) Enrichment of propanoate metabolism-associated genes across 40 tissues and 81 cell subclasses, utilizing mRNA expression data from GTEx and the Human Protein Atlas (HPA), normalized as Transcripts Per Million (nTPM). (**C**) Network diagram visualizing interactions among propanoate metabolism genes across various cell types and tissues, highlighting the complex interplay and shared metabolic pathways. Data integration from GTEx and the HPA reveals significant connections, particularly between hepatocytes and kidney proximal tubular cells, suggesting cross-organ metabolic interactions. (**D**) Kaplan–Meier survival curves for HCC patients based on liver-specific gene expression as indicated. The plot displays survival rate of 365 HCC patients from the TCGA-LIHC dataset, stratified into high and low expression groups based on liver-specific genes involved in propanoate metabolism. The expression cutoff was determined using FPKM (Fragments Per Kilobase of transcript per Million mapped reads) values that maximized survival differences between groups. Statistical significance was assessed via log-rank test. (**E**) Hazard ratios for liver-specific genes associated with HCC prognosis, based on data from 365 patients in the TCGA-LIHC cohort. High expression levels of genes such as *ALDH2* (HR = 1.87, 95% CI: 1.18–2.90) and *ALDH1B1* (HR = 1.64, 95% CI: 1.06–2.50) correlate with a significantly increased risk of poor prognosis. (**F**) Chromatographic analysis and differential metabolite profiling in HCC and HCC risk group. Top panel shows the logarithmic intensity of metabolites across retention times, identifying peaks significantly elevated in the HCC patients compared to the HCC risk group. The bottom panel highlights the fold-change in metabolite concentrations, pinpointing significant differences between the two groups. (**G**) Chromatographic intensity corresponding to the serum concentration of key metabolites—toluene, p-xylene, m-xylene, o-xylene, and 2-ethylhexanol—across liver disease states: HCC, HCV, HBV, and LC. Inset: Schematic diagram showing the relation of precursors and byproduct with propionyl-CoA in propanoate metabolism. Statistical comparisons between groups demonstrate significant differences (*p* values ranging from 5.46 × 10⁻⁶ to 4.00 × 10⁻¹²). (**H**) Comparison of metabolite concentrations across cancer types: HCC, PC, CRC, and KC. While toluene and xylene isomers show limitations in distinguishing HCC from PC, 2-ethylhexanol demonstrates a distinctive pattern with significant depletion specifically in HCC compared to non-target cancer types (*p* values ranging from 1.48 × 10⁻⁴ to 2.95 × 10⁻¹³). *p* values were obtained by t-tests in (F-H): *, **, ***, and **** indicate *p* < 0.05, *p* < 0.01, *p* < 0.001, and *p* < 0.0001, respectively

Further verification of these findings using data from the Human Protein Atlas (HPA) provided strong corroboration, revealing that 39.4% (13/33 genes) were predominantly expressed in the liver (Supplementary Figure S1-S2). Such liver-specific enrichment emphasizes the unique metabolic regulation occurring within hepatocytes, supporting the feasibility of these genes as diagnostic biomarkers uniquely indicative of liver pathology. We then conducted enrichment network analysis to further delineate tissue-specific associations. Intriguingly, our enrichment network analysis revealed distinct clustering of hepatocytes with kidney proximal tubular cells, indicating potential cross-organ metabolic connections within the propanoate metabolism framework (Fig. 1C and Supplementary Figure S3). While the biological significance of this association remains to be experimentally validated, these results raise the possibility of metabolic crosstalk between liver and kidney in regulating propanoate metabolism. Notably, despite this observed link, survival analysis using the TCGA-LIHC cohort confirmed that liver-specific genes such as *ALDH2* and *ABAT* exhibited the strongest prognostic impact, with 26.6% and 19.9% survival differences at 65 months, respectively, compared to much smaller effects observed for non-LPM genes (e.g., *PCCA*) (Supplementary Table S1). These findings emphasize that the prognostic power and pathophysiological relevance of propanoate metabolism in HCC are predominantly driven by liver-specific genes.

Leveraging clinical and genomic data from The Cancer Genome Atlas-Liver Hepatocellular Carcinoma (TCGA-LIHC) cohort (*n* = 365), we further investigated the prognostic relevance of identified LPM gene signatures. Patients stratified into a high-risk LPM group had significantly shorter overall survival compared to a low-risk LPM group (*p* < 0.001) (Fig. 1D, Supplementary Table S1). Notably, two liver-specific genes, *ALDH2* and *ALDH1B1*, emerged as highly significant prognostic indicators, presenting hazard ratios of 1.87 (95% confidence interval [CI]: 1.18–2.90) and 1.64 (95% CI: 1.06–2.50), respectively (Fig. 1E). These results highlight the prognostic utility of liver-specific metabolic markers, particularly *ALDH2* and *ALDH1B1*, for accurately stratifying risk and predicting outcomes in patients with HCC.

To evaluate the diagnostic efficacy of specific metabolites associated with propanoate metabolism for HCC diagnosis, we performed targeted metabolomic profiling with isotope-labeled standards using serum samples collected from 273 HCC patients (Supplementary Table S2-S3). Additionally, we included serum samples from various risk groups, such as those diagnosed with HBV, HCV, LC, and several non-target cancers, specifically colorectal cancer (CRC), kidney cancer (KC), and pancreatic cancer (PC), collected across six different hospitals. Our targeted metabolomic analysis was conduced via gas chromatography-mass spectrometry (GC-MS), and highlighted notable alterations in several metabolites implicated in propanoated metabolism pathways (Supplementary Figure S4). We observed significantly elevated serum concentrations of toluene and xylene isomers in HCC patients compared to risk groups, indicating their potential utility in distinguishing HCC through metabolomic profiling (Fig. 1F-G). Particularly, toluene levels were significantly higher in HCC patients compared to HCV, HBV, and LC (*p* values ranging from 3.90 × 10⁻¹² to 4.00 × 10⁻¹²), and xylene isomers were markedly elevated in HCC patients (*p* values ranging from 5.46 × 10⁻⁶ to 4.23 × 10⁻⁹). Conversely, we found a substantial reduction in 2-ethylhexanol in HCC patients versus HCV (*p* = 4.95 × 10⁻⁴), HBV (*p* = 1.22 × 10⁻⁶), and LC (*p* = 6.32 × 10⁻¹²), suggesting an increased metabolic consumption unique to liver malignancies. Since toluene and xylene isomers are propanoate metabolism precursors, and 2-ethylhexanol is a byproduct of β-oxidation in propanoate metabolism, serum concentration of those metabolites should closely correspond to diagnostic accuracy of HCC.

In addition, toluene and xylene isomers were upregulated in CRC and KC over HCC (Fig. 1H), while these precursors showed limitations distinguishing HCC from PC, likely due to similar metabolic alterations [5]. However, the decreaed serum level of 2-ethylhexanol robustly differentiated HCC from CRC (*p* = 2.95 × 10⁻¹³), KC (*p* = 8.92 × 10⁻⁷), and PC (*p* = 1.48 × 10⁻⁴), emphasizing its remarkable specificity as a diagnostic biomarker for HCC. Despite positive correlation between 2-ethylhexanol and increased toluene and xylene isomers (Supplementary Figure S5), significantly lower 2-ethylhexanol levels in HCC likely result from increased consumption characteristic of liver-specific propanoate metabolism [6]. Decreased 2-ethylhexanol levels achieved perfect discrimination from HCC risk group (AUC = 1.000) and excellent diagnostic performance for distinguishing HCC from non-target cancers (AUC = 0.904), emphasizing its remarkable specificity as a diagnostic biomarker for HCC (Supplementary Figure S6). Given that 2-ethylhexanol is directly linked to lipid peroxidation through propanoate metabolism, this biomarker also opens novel avenues for therapeutic intervention, particularly in drug development targeting ferroptosis, a cell-death mechanism highly relevant to cancer biology [7].

From a clinical perspective, 2-ethylhexanol shows strong potential to improve HCC diagnosis and prognosis. Unlike alpha-fetoprotein (AFP), which has limited sensitivity and specificity, 2-ethylhexanol achieved superior performance (AUC = 1.000 vs. high-risk groups; AUC = 0.904 vs. non-target cancers). Rather than replacing current methods, these metabolite markers can complement AFP and imaging, enhancing diagnostic accuracy, particularly in early-stage or borderline cases. Moreover, when combined with prognostic liver-specific genes such as *ALDH2* and *ALDH1B1*, these metabolites may form multi-omics panels for more precise risk stratification and personalized treatment planning. While acknowledging that our study employs a cross-sectional metabolite analysis, limiting the ability to assess temporal metabolite changes during disease progression, our findings nevertheless significantly contribute to the emerging field of metabolomics-based cancer diagnostics. Prospective longitudinal studies assessing diagnostic performance over disease progression and diverse patient cohorts are warranted to fully validate these biomarkers in clinical practice (Supplementary Note S3 and S4).

In conclusion, our integrated binary omics and metabolomic profiling approach has effectively identified liver-specific metabolic alterations that significantly enhance the precision of serum-based HCC diagnosis and prognosis. The combined assessment of liver-specific propanoate metabolism genes and metabolite markers, particularly 2-ethylhexanol, demonstrates substantial promise as a robust diagnostic and prognostic tool, potentially transforming clinical approaches to HCC detection and treatment.

## Supplementary Information

Below is the link to the electronic supplementary material.

Supplementary Material 1

## Data Availability

Data are available from the corresponding authors upon reasonable request.

## Abbreviations

HBV: Hepatitis B virus
HCV: Hepatitis C virus
LC: Liver cirrhosis
HCC: Hepatocellular carcinoma
CRC: Colorectal cancer
KC: Kidney cancer
PC: Pancreatic cancer
LPM: Liver-specific propanoate metabolism
TCGA-LIHC: The cancer genome atlas liver hepatocellular carcinoma
HR: Hazard ratio
CI: Confidence interval
