# Supplementary material for: Liver-specific propanoate metabolism-derived 2-ethylhexanol as a novel biomarker for precise diagnosis and prognosis of hepatocellular carcinoma
Source: Biomark Res. 2025 Oct 21;13:129. doi: 10.1186/s40364-025-00842-7 (PMC12539108; doi:10.1186/s40364-025-00842-7)
Supplement: Supplementary file 1 — Supplementary Material 1 [file 40364_2025_842_MOESM1_ESM.pdf]

Supplementary Information for

Liver-specific propanoate metabolism-derived 2-ethylhexanol as a novel biomarker for precise diagnosis and prognosis of hepatocellular carcinoma

Dongyong Lee[1], Jinhee Mun[1], Jihyun Lee[1], Chan Jang[2], Geumjong Song[3], Jonghyuk Yun[3], Kwangseock Kim[4], Taewan Kim[4], Seob Jeon[5,*], Jinmyoung Joo[1,6,7,*]

[1] Department of Biomedical Engineering, Ulsan National Institute of Science and Technology, Ulsan, 44919, Republic of Korea

[2] Department of Computer Science and Engineering, Ulsan National Institute of Science and Technology, Ulsan, 44919, Republic of Korea

[3] Department of Surgery, Division of Gastrointestinal Surgery, College of Medicine, Soonchunhyang University Cheonan Hospital, Cheonan 31151, Republic of Korea

[4] Future Innovation Medical Research Center, Soonchunhyang University Cheonan Hospital, Cheonan 31151, Republic of Korea

[5] Department of Gynecology, College of Medicine, Soonchunhyang University Cheonan Hospital, Cheonan 31151, Republic of Korea

[6] Graduate School of Health Science and Technology, Ulsan National Institute of Science and Technology, Ulsan, 44919, Republic of Korea

[7] Center for Genomic Integrity, Institute for Basic Science, Ulsan, 44919, Republic of Korea

[*]Correspondence:

Seob Jeon, sjeon4595@gmail.com

Jinmyoung Joo, jjoo@unist.ac.kr

Supplementary Note S1. Tissue-specific expression profile of propanoate metabolism genes and hepatocyte-enriched metabolic signature

Under normal physiological conditions, propanoate metabolism in hepatocytes follows a well-defined biochemical pathway where propionyl-CoA is converted to methylmalonyl-CoA by propionyl-CoA carboxylase, then to succinyl-CoA by methylmalonyl-CoA mutase, ultimately feeding into the tricarboxylic acid cycle [1, 2]. This metabolic cascade plays essential roles in fatty acid β-oxidation, amino acid catabolism particularly of valine, isoleucine, threonine, and methionine, cholesterol side-chain oxidation, and gut microbiota-derived propionate metabolism [3, 4]. Various acyl-CoA dehydrogenases and aldehyde dehydrogenases, including ALDH2 and ALDH1B1, participate in processing metabolic intermediates and preventing toxic metabolite accumulation [5, 6]. Our comprehensive analysis across 40 normal tissues and 81 cell subclasses using GTEx and HPA databases demonstrated that propanoate metabolism is not uniformly distributed but rather exhibits marked tissue specificity (Supplementary Figure S1) [7, 8]. The liver emerged as the primary site, with 45.5% (15/33) of pathway genes showing elevated expression compared to other tissues, validated through both databases with 39.4% concordance (Supplementary Figure S2) [9, 10]. While propanoate metabolism occurs in multiple tissues including kidney, pancreas, and colon, the liver demonstrates the highest concentration of pathway-specific genes and enzymatic capacity (Figure 1B) [11, 12]. This tissue distribution pattern reflects the liver's central role in metabolic homeostasis and its unique capacity for processing short-chain fatty acids and amino acid metabolites, distinguishing it from other metabolically active organs [13-15]. The gut microbiota contributes significantly to propionate production through three distinct pathways, further emphasizing the systemic nature of propanoate metabolism [16-18].

Supplementary Note S2. Prognostic impact of liver-specific propanoate metabolism gene signatures in hepatocellular carcinoma

The prognostic significance of LPM genes extends beyond mere association, as demonstrated by our cumulative survival analysis which revealed striking differences between high and low expression groups (Supplementary Table S1). For ALDH2, patients with high expression showed only 26.2% survival at 65 months compared to 52.8% for low expression, representing a 26.6 percentage point difference (Supplementary Table S1). Similarly, ABAT demonstrated a 19.9 percentage point survival difference with 32.5% versus 52.4% survival at 65 months (Supplementary Table S1). In contrast, non-LPM genes such as PCCA showed minimal survival differences of only 5.4 percentage points, emphasizing the specific prognostic value of liver-enriched genes (Supplementary Table S1). These genes encode key enzymes in aldehyde metabolism and amino acid catabolism, and their dysregulation likely contributes to the accumulation of toxic metabolic intermediates that promote hepatocarcinogenesis (Figure 1D). The coordinated upregulation of LPM genes, particularly ALDH2 (HR=1.87, 95% CI: 1.18-2.90) and ABAT (HR=1.39, 95% CI: 0.89-2.20), suggests they drive disease progression rather than merely reflecting it (Figure 1E). This is consistent with previous reports demonstrating that decreased propionyl-CoA metabolism facilitates metabolic reprogramming in HCC.

Supplementary Note S3. 2-Ethylhexanol as a highly specific non-invasive diagnostic biomarker for hepatocellular carcinoma

The identification of 2-ethylhexanol as a highly specific HCC biomarker represents a significant advancement in non-invasive diagnostics that addresses critical limitations of current methods. Our metabolomic profiling of 273 patients (Supplementary Table S3), with rigorous protocols to preserve volatile compound integrity including immediate serum separation, -80°C storage, and direct headspace GC-MS analysis with internal standards, revealed that 2-ethylhexanol levels were dramatically reduced in HCC compared to high-risk groups (Figure 1F-G). The diagnostic performance of 2-ethylhexanol substantially exceeds that of alpha-fetoprotein (AFP), the current clinical standard which typically shows only 60-70% sensitivity at 90% specificity. Our comprehensive ROC analysis demonstrated perfect discrimination from high-risk groups with AUC of 1.000 and 100% accuracy (Supplementary Figure S5A), achieving p-values of 6.32×10^{-12} for HCC versus cirrhosis, 1.22×10^{-6} for HCC versus HBV, and 4.95×10^{-4} for HCC versus HCV (Supplementary Table S4). Furthermore, 2-ethylhexanol showed high specificity for HCC versus other cancers with AUC of 0.904 and 85.3% accuracy (Supplementary Figure S5B), including kidney cancer ($p=8.92\times10^{-7}$), pancreatic cancer ($p=1.48\times10^{-4}$), and colorectal cancer ($p=2.95\times10^{-13}$) (Supplementary Table S5). The paradoxical decrease in 2-ethylhexanol despite elevated precursors such as toluene and xylene isomers (Supplementary Figure S3) suggests increased consumption characteristic of liver-specific metabolic reprogramming (Figure 1H). This pattern aligns with enhanced β-oxidation and altered fatty acid metabolism previously described in HCC, reflecting the unique metabolic demands of hepatocellular transformation (Supplementary Figure S4).

Supplementary Note S4. Clinical translation potential, study limitations, and future research directions

The clinical utility of our findings extends beyond diagnostic applications, offering multiple practical advantages over existing methods. The use of standard GC-MS technology with established protocols (Supplementary Table S6) facilitates immediate clinical translation without requiring specialized equipment. The small sample volume requirement of only 200 μL serum and rapid turnaround time of 30-60 minutes make this approach practical for routine clinical use. Our biomarker panel could serve multiple clinical purposes including first-line screening for high-risk populations with cirrhosis or chronic hepatitis, disease progression monitoring from cirrhosis to HCC through metabolic changes, clarification of equivocal imaging findings when conventional methods are inconclusive, therapeutic response assessment through metabolic normalization, and risk stratification for intensive surveillance based on LPM gene expression profiles (Supplementary Table S1, Figure 1D-E). Furthermore, the mechanistic insights into propanoate metabolism dysfunction open therapeutic avenues. The identification of specific metabolic vulnerabilities, particularly in aldehyde metabolism and propanoate processing, suggests potential targets for metabolic intervention strategies.

We acknowledge several limitations that should be addressed in future studies, though these do not diminish the transformative potential of our findings. Our cross-sectional design limits assessment of temporal metabolite changes during disease progression, and longitudinal studies tracking metabolite profiles from cirrhosis through HCC development would provide valuable insights into the timing and sequence of metabolic alterations. While we identified metabolic connections between hepatocytes and kidney proximal tubular cells through network analysis (Figure 1C), functional validation through metabolic tracing studies or organ-specific knockout models is needed to confirm actual metabolic cooperation. Although our multi-center design provides robust initial validation and the exceptional performance metrics with perfect 100% accuracy for HCC versus high-risk groups (Supplementary Figure S5A) warrant immediate attention, prospective validation in independent cohorts, particularly in diverse ethnic populations and different etiological backgrounds such as alcohol-related, NASH, and viral hepatitis, is essential before clinical implementation. The precise biochemical basis for 2-ethylhexanol depletion in HCC remains incompletely understood, and detailed metabolic flux analysis and isotope tracing studies would clarify the specific pathways and enzymatic steps involved in its altered metabolism (Supplementary Figure S3, S4). Future studies should also

evaluate the combination of 2-ethylhexanol measurement with conventional imaging to develop optimal diagnostic algorithms, though given its perfect discrimination from high-risk groups, 2-ethylhexanol could potentially serve as a standalone first-line test.

In conclusion, our integrated multi-omics approach has identified liver-specific propanoate metabolism alterations as a defining metabolic feature of HCC, with 2-ethylhexanol emerging as a superior diagnostic biomarker compared to current clinical standards (Supplementary Figure S5). The exceptional diagnostic performance with AUC of 1.000 for distinguishing HCC from high-risk groups (Supplementary Figure S5A), combined with the strong prognostic significance of LPM genes showing progressive survival deterioration from 61.5% to 26.2% at 65 months for ALDH2 high expression versus 83.3% to 52.8% for low expression (Supplementary Table S1), provides both immediate clinical applications and fundamental insights into HCC pathogenesis. The tissue-specific nature of these metabolic alterations, particularly the coordinated dysregulation of liver-enriched genes (Supplementary Figure S1-S2), emphasizes the unique metabolic reprogramming that characterizes hepatocellular transformation. Future efforts should focus on prospective validation in diverse populations, mechanistic elucidation of metabolic flux alterations, and development of integrated diagnostic algorithms that leverage these metabolic signatures for improved early detection and ultimately better patient outcomes in this aggressive malignancy.

Supplementary Note S5. Technical Methodology for Volatile Metabolite Preservation and Quantification in Serum Samples

The identification of 2-ethylhexanol as a highly specific HCC biomarker represents a breakthrough advancement in non-invasive diagnostics that fundamentally addresses the critical limitations of current methods. However, the detection of volatile metabolites poses unique technical challenges that have hindered previous discovery efforts. Our metabolomic profiling of 273 patients (Supplementary Table S2) therefore employed carefully designed protocols specifically engineered to preserve volatile compound integrity, a critical technical achievement that distinguishes our study from previous metabolomic investigations in HCC. Blood samples were collected in SST tubes and immediately centrifuged at 1500 × g at 4 °C for 10 minutes, with serum separation conducted within one hour of blood collection to prevent metabolite degradation. Most critically, the immediate transfer to -80 °C storage within sealed, gas-impermeable containers was the cornerstone of our volatile preservation strategy, as this ultra-low temperature fundamentally prevents volatilization by maintaining these compounds far below their phase transition temperatures, effectively trapping volatile metabolites in a state where evaporation is thermodynamically impossible. Complementing this temperature control, our quality control system included adding internal standard of 1 μg/mL Chlorobenzene-d5 to each sample for quantification accuracy, evaluating matrix blanks and matrix spikes for each analytical batch to ensure no contamination or loss, performing headspace extraction with controlled agitation at exactly 90 °C for 10 minutes to ensure complete volatile recovery without thermal degradation (Supplementary Table S6), and maintaining a hermetically sealed gas-tight analytical system to prevent any volatile loss during analysis. We monitored recovery rates throughout the study, achieving consistent recoveries above 95 % for all target metabolites (Supplementary Figure S3).

Supplementary Methods

Ethics

This research was approved by the Institutional Review Board (IRB) of Ulsan National Institute of Science and Technology (UNISTIRB-20-07-C) and Soonchunhyang University Cheonan Hospital (SCH-IRB-2023-10-030).

Patient samples

This research was part of a multicenter, retrospective cohort analysis conducted within the metabolite-based pan-cancer project, aiming to understand the progression from HCC risk group (LC, HBV, HCV) to HCC. Additionally, serum samples from patients diagnosed with four types of cancer were collected for comparative analysis. A total of 273 patient samples (Supplementary Table S2) were gathered from six clinical institutes across the Republic of Korea: Jeonbuk National University Hospital, Ajou University Hospital, Gangwon National University Hospital, Inje University Busan Paik Hospital, Kyungpook National University Hospital, and Chungbuk National University Hospital.

RNA expression analysis of 40 tissues types and 81 cell subclasses

mRNA expression data were obtained from GTEx (https://gtexportal.org) and the Human Protein Atlas (HPA, https://www.proteinatlas.org). Values were normalized using normalized transcripts per million (nTPM). The Human Protein Atlas (HPA) is a publicly available database that provides single-cell RNA sequencing (scRNA-seq) data for single cell types across 31 human tissues. HPA datasets were retrieved from the Single Cell Expression Atlas, the Human Cell Atlas, the Gene Expression Omnibus, the Allen Brain Map, the European Genome-phenome Archive, and the Tabula Sapiens. Transcript profiling was conducted using a combination of two transcriptomics datasets (HPA and GTEx) from 50 different human normal tissue types.

Evaluation of specificity

Normalized expression (nTPM) value for each tissue type was used for the classification of all genes according to the tissue-specific expression into two different categories, based on specificity or distribution. (1) Tissue enriched: At least four-fold higher mRNA level in a particular tissue compared to any other tissue. (2) Group enriched: At least four-fold higher average mRNA level in a group of 2-

5 tissues compared to any other tissue. (3) Tissue enhanced: At least four-fold higher mRNA level in a particular tissue compared to the average level in all other tissues

Metabolite profiling analysis

Blood collection and sample preparation

At all participating hospitals, blood collection and serum preparation were performed using the same protocol by trained clinical staffs. Venous blood samples (10 mL) were drawn from each participant via venipuncture and collected in SST tubes. The serum samples were centrifuged at 1500 × g at 4 °C for 10 minutes. Serum separation was conducted within 1 hour of blood sample collection, and the serum was immediately stored at −80 °C. Metabolic profiling was conducted in a blinded manner. Each serum sample was labeled with a unique barcode identifier to ensure that laboratory researchers were blinded to the clinical information. An equal amount of analytical samples (200 μL) was mixed with an internal standard (1 μg/mL of Chlorobenzene-d5). To assess the quality of the analyzed samples, matrix blanks (200 μL of methanol) and matrix spikes with the internal standard (200 μL mixture of methanol and 1 μg/mL Chlorobenzene-d5) were evaluated for each batch of the analysis.

External and internal standard

Analytical grade methanol, 4-bromofluorobenzene, toluene, p-xylene, m-xylene, o-xylene, 2-ethylhexanol, isotopically labeled analytical standards (Chlorobenzene-d5), and Gibco™ FBS were purchased from Sigma-Aldrich (Gillingham, UK).

Chemical extraction

Serum extraction conditions evaluated included: (i) extraction temperature and time; (ii) sample acidification; (iii) sample volume; (iv) sample dilution; and (v) headspace versus immersive analysis. Baseline conditions were as follows: 200 μL undiluted, unacidified serum samples were extracted at 90 °C for 15 minutes using headspace analysis.

GC-MS (gas chromatography-mass spectrometry with headspace injection)

The analysis of toluene, *p*-xylene, *m*-xylene, *o*-xylene, and 2-ethylhexanol was carried out using

a Thermo Fisher Trace 1310 gas chromatographic system coupled to a Thermo Fisher ISQ 7000 mass spectrometer equipped with electron ionization (EI). Chromatographic analysis was conducted in splitless mode. The GC column used was a Thermo Fisher TraceGold TG-624-SilMs capillary column (30 m × 0.32 mm × 0.18 μm, catalog no. 26059-3390). A Thermo Fisher Linergold splitless single taper (4 × 6.5 × 78.5 mm) with a glass liner was used in the inlet for injection. Headspace extraction with an agitator was employed to capture the metabolites, with each vial agitated at 90 °C for 10 minutes. Helium was used as the carrier gas at a flow rate of 1 mL/min. The oven gradient was set to start at 40 °C and increased to 280 °C at a rate of 10 °C/min, holding for 1 minute. The total run time was 30 minutes. The MS transfer line was maintained at 280 °C throughout the run (Detailed parameters are described in Supplementary Table S6).

Identification and quantification of serum metabolites

Data was collected from range of 45-450 m/z (masss-to-charge ratio) with a scan mode. Data analysis was performed using TraceFinder General Quant 5.1 software and Freestyle 1.7 with NIST library.

Metabolomic data analysis and statistics

GC/MS data were log-transformed, and the median expression of each metabolite was subtracted for downstream analysis. For differential expression analyses, fold change was computed by taking the exponential of the difference between the median expressions of the two groups (HCC vs. non-target). Some subclasses had a small number of samples, resulting in inaccurate statistical tests. Therefore, three statistical tests (Anderson-Darling test, WiHCCoxon rank sum test, Kolmogorov-Smirnov test) were used, and metabolites with p-values ≤ 0.05 in all three tests were selected as metabolites of interest.

Statistical analysis

For Kaplan-Meier survival analysis, patients were divided into two groups, and the association between prognosis (survival) and gene expression (FPKM) was examined. The best expression cut-off refers to the FPKM value that yields the maximal difference in survival between the two groups at the lowest log-rank p-value. The best expression cut-off was selected based on survival analysis. Cox

regression models were used for univariate and multivariate analyses of prognostic factors, with p-values < 0.05 regarded as statistically significant (Supplementary Table S1).

For metabolite profiling, statistical significance was determined by p value (* $p < 0.05$; ** $p < 0.01$; *** $p < 0.001$; **** $p < 0.0001$). Differences between HCC and other groups were analyzed using one-way ANOVA, Student's t-test, or Mann-Whitney U test. Correlations between chemicals were analyzed using Spearman (ρ), Kendall (τ), and Pearson (r) rank correlation coefficients. Statistical analyses were conducted using R 3.4.2 (Supplementary Tables S3 and S4).

Supplementary Figure S1. Enrichment analysis of propanoate metabolism gene expression across (A) tissue types and (B) cell subclasses.

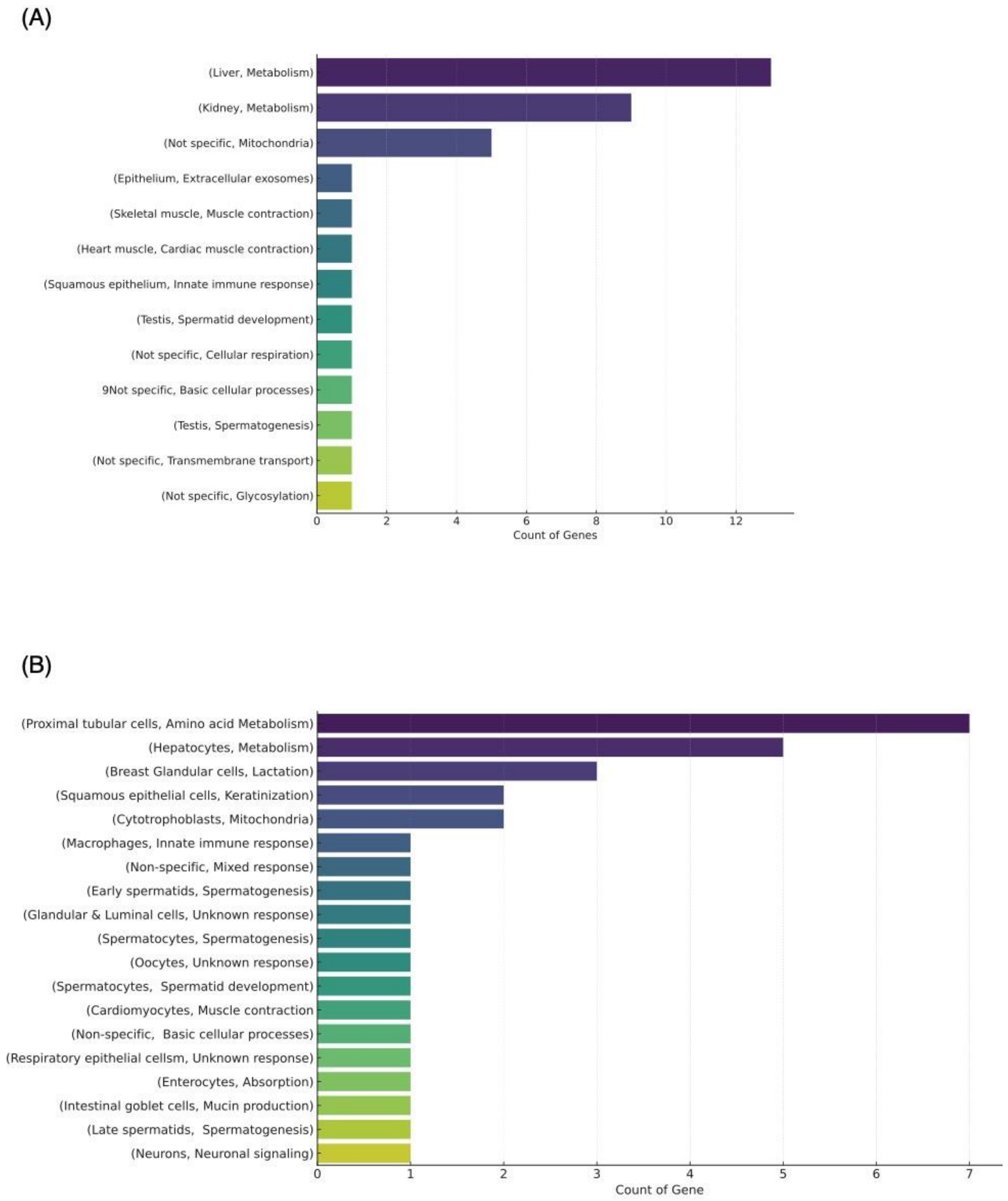

Supplementary Figure S2. Venn diagram of liver-specific and propanoate metabolism genes. (A) A venn diagram illustrates the overlapping significantly identified pathways resulting from the comparison of propanoate metabolism-dependent regulated pathways in a common tissue and liver-tissue-specific context. (B) Propanoate metabolism-specific genes and selected LPM genes, corresponding to the venn diagram in (A).

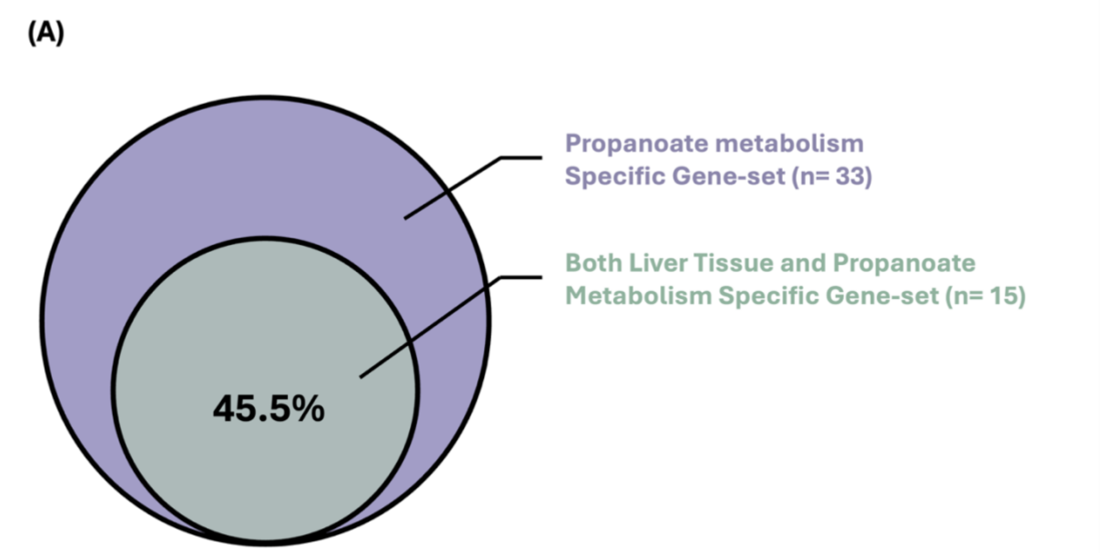

(B)

Propanoate metabolism specific gene-set

Gene
ALDH2
ALDH1A3
EHHADH
ACACB
ALDH6A1
ABAT
SUCLA2
SUCLG2
ALDH9A1
MCEE
HADHA
ALDH3A1
LDHC
HIBCH
LDHB
ACSS2
ALDH1B1
MUT
MLYCD
LDHA
LDHAL6A
ACAT2
SUCLG1
ACADM
ALDH7A1
LDHAL6B
ACSS1
PCCA
ACAT1
ALDH3A2
PCCB
ACACA
ECHS1

Selected LPM genes

Gene
ALDH2
EHHADH
ALDH6A1
ABAT
SUCLG2
ALDH1B1
MUT
ACAT2
ACADM
ALDH7A1
ACAT1
ALDH3A2
PCCB
ACACA
ECHS1

Supplementary Figure S3. Precursors of propanoate metabolism pathway. The precursors (*p*-, *o*-, *m*-Xylene, and Toluene) in propanoate metabolism is selected by KEGG pathway.

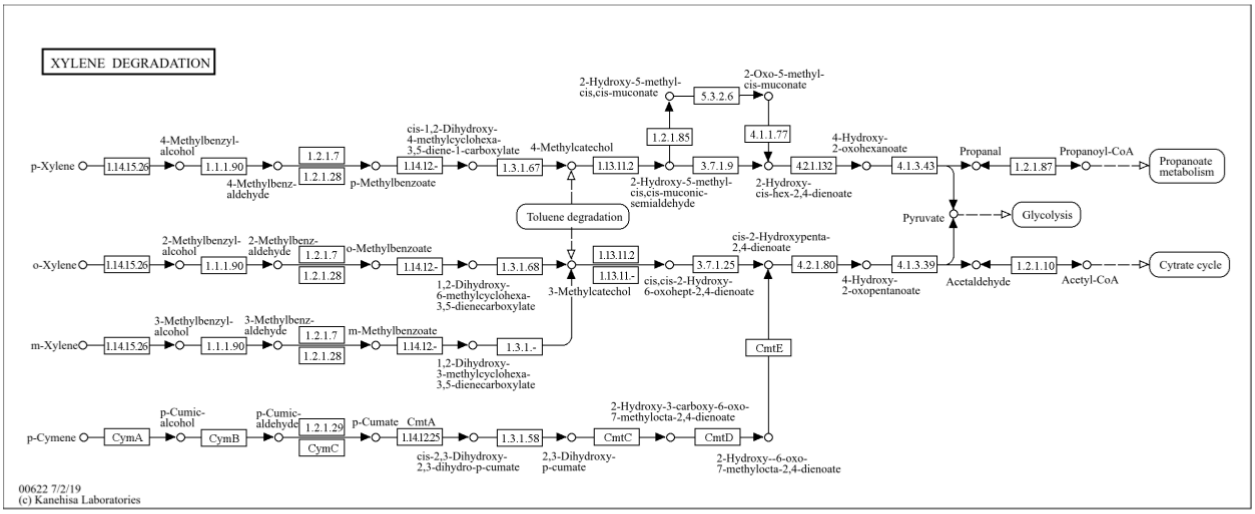

Supplementary Figure S3. Network diagram visualizing interactions among propanoate metabolism genes across various cell types and tissues (enlarged from Figure 1C). Data integration from GTEx and the HPA reveals significant connections, particularly between hepatocytes and kidney proximal tubular cells, suggesting cross-organ metabolic interactions. This analysis highlights the complex interplay and shared metabolic pathways.

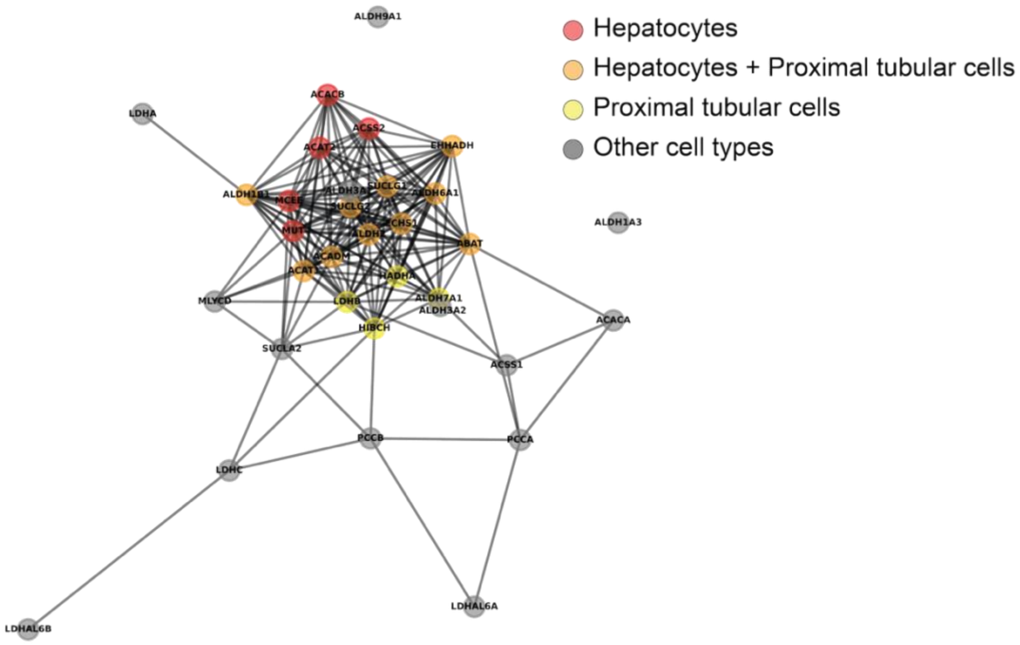

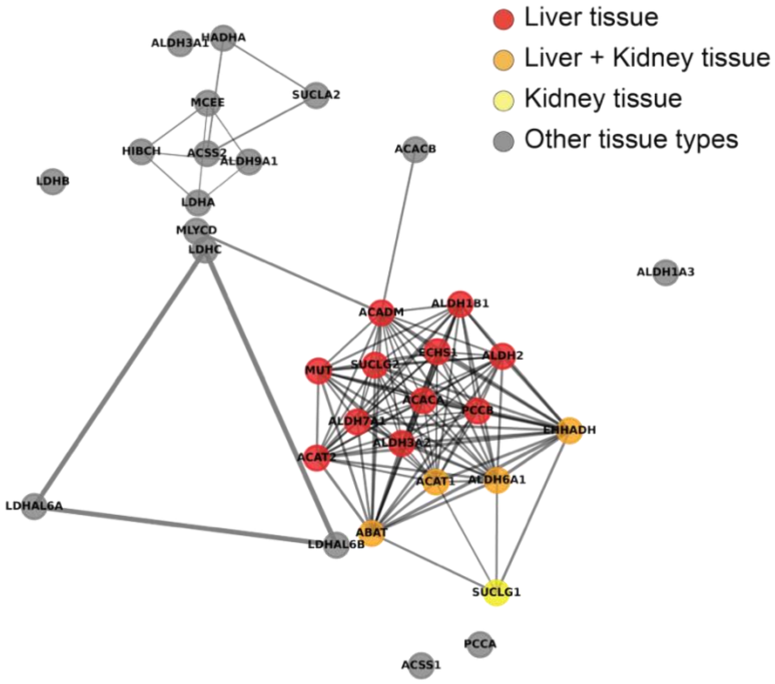

Supplementary Figure S5. Scatter plots of pairwise comparison between profiled serum metabolites. This pairwise matrix shows the Pearson correlation coefficients between each pair of variables. Detailed *p*-value and statistical coefficient are in Supplementary Table S5.

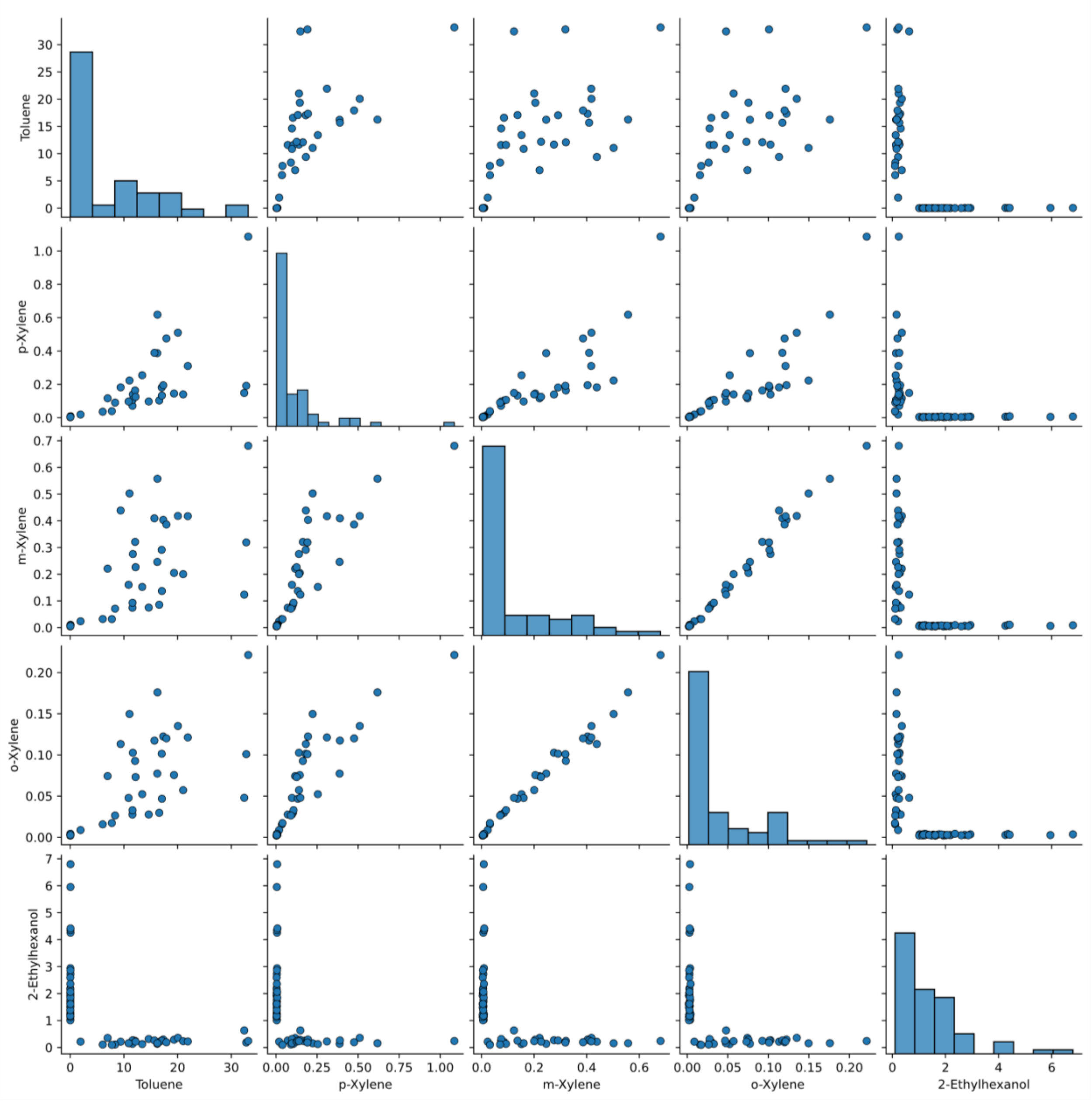

Supplementary Figure S6. Receiver operating characteristic (ROC) curves for metabolite-based discrimination of HCC. (A) ROC curves demonstrating the diagnostic performance of serum metabolites (toluene, p-xylene, m-xylene, o-xylene, and 2-ethylhexanol) in distinguishing HCC patients from the HCC risk group (HBV, HCV, and LC). 2-Ethylhexanol achieved perfect discrimination with AUC = 1.000 and ACC = 1.000. (B) ROC curves showing the diagnostic performance of the same metabolites in differentiating HCC from non-target cancers (CRC, KC, and PC). 2-Ethylhexanol demonstrated the highest diagnostic accuracy with AUC = 0.904 and ACC = 0.853.

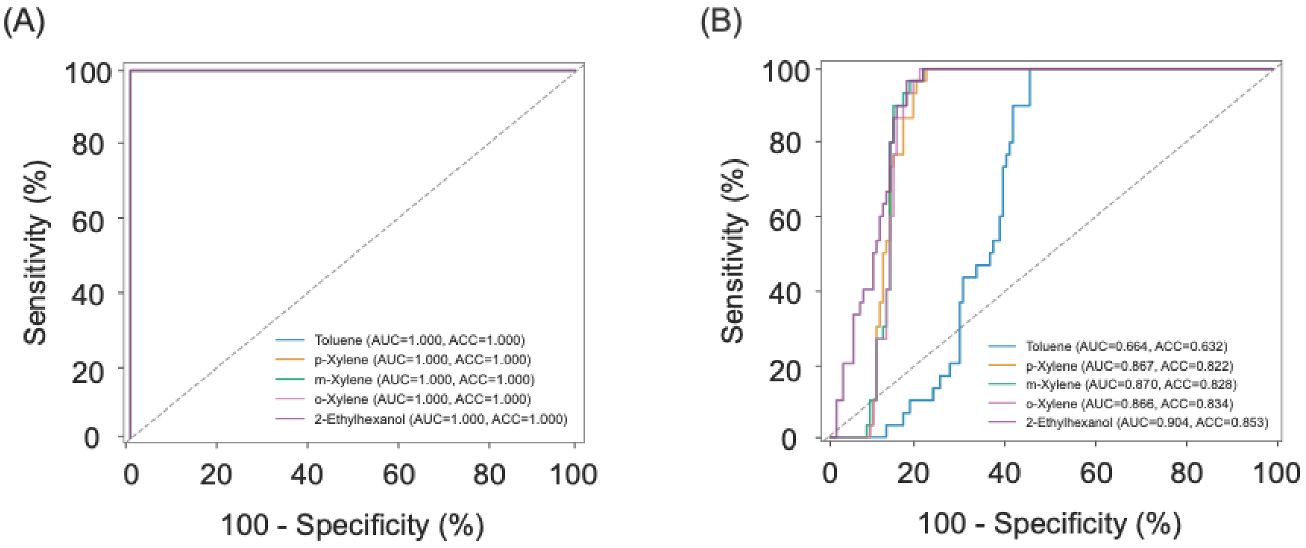

Supplementary Table S1. Cumulative survival rates of HCC patients stratified by LPM and Non-LPM genes.

	**LPM gene**				**Non-LPM gene**			
	ABAT		***ALDH2***		***ALDH3A1***		***PCCA***	
**(Month)**	**High Risk**	**Low Risk**	**High Risk**	**Low Risk**	**High Risk**	**Low Risk**	**High Risk**	**Low Risk**
**0**	100.0%	100.0%	100.0%	100.0%	100.0%	100.0%	100.0%	100.0%
**16**	62.1%	84.5%	61.5%	83.3%	74.5%	81.2%	75.5%	82.1%
**32**	44.6%	71.7%	42.0%	70.3%	63.0%	65.0%	60.3%	69.5%
**48**	39.7%	61.0%	30.6%	61.8%	50.5%	58.7%	50.3%	62.0%
**65**	32.5%	52.4%	26.2%	52.8%	44.3%	48.4%	44.7%	50.1%

Supplementary Table S2. Characteristics of subjects in TCGA-LIHC cohort.

**Variables**	**Patients with HCC** (*n* = 365)
**Age** (years), mean ± SD	59.86 ± 13.09
**Gender**, *n* (%)	
Male	246 (67.4)
Female	119 (32.6)
**Race**, *n* (%)	
White	182 (50.0)
Asian	155 (42.58)
Black or african american	17 (4.67)
NA	10 (2.75)
**Pathologic stage** (AJCC 8th) , *n* (%)	
i	170 (46.6)
ii	84 (23.0)
iii	3 (0.8)
iiia	63 (17.3)
iiib	8 (2.2)
iiic	9 (2.5)
iv	1 (0.3)
iva	1 (0.3)
ivb	2 (0.5)
NA	24 (6.6)

Abbreviations: AJCC, American Joint Committee on Cancer; HCC, Hepatocelluar carcinoma; NA, not available; SD, standard deviation.

Supplementary Table S3. Patient samples collected for serum metabolite profiling.

**Disease**	**Participant patients** (n = 273)
Hepatocelluar carcinoma (HCC)	30
Hepatitis type B (HBV)	10
Hepatitis type C (HCV)	10
LC	
Hepatitis type B induced LC (HBV)	17
Hepatitis type C induced LC (HCV)	3
Cholecystitis	50
Gallbladder cancer (GC)	6
Cholangiocarcinoma	4
Pancreatic cancer (PC)	43
Colorectal cancer (CRC)	50
Kidney cancer (KC)	50

Supplementary Table S4. Statistial analysis using t-test and ANOVA for comparison between HCC and HCC risk group.

Metabolite	t-test			ANOVA
	vs. HCV	vs. HBV	vs. LC	
Toluene	4.00×10^{-12}	3.90×10^{-12}	3.92×10^{-12}	5.58×10^{-18}
p-Xylene	6.02×10^{-6}	5.59×10^{-6}	5.46×10^{-6}	6.79×10^{-7}
m- Xylene	1.37×10^{-8}	1.31×10^{-8}	1.17×10^{-8}	2.24×10^{-11}
o- Xylene	4.55×10^{-9}	4.77×10^{-9}	4.23×10^{-9}	3.48×10^{-12}
2-Ethylhexanol	4.95×10^{-4}	1.22×10^{-6}	6.32×10^{-12}	1.24×10^{-16}

Supplementary Table S5. Statistial analysis using t-test and ANOVA for comparison between HCC with other cancers.

Metabolite	t-test			ANOVA
	vs. CRC	vs. KC	vs. PC	
Toluene	1.43×10^{-14}	2.18×10^{-4}	9.83×10^{-1}	2.77×10^{-12}
p-Xylene	8.42×10^{-16}	8.44×10^{-11}	2.77×10^{-1}	8.75×10^{-20}
m-Xylene	4.30×10^{-24}	1.17×10^{-16}	3.11×10^{-1}	8.00×10^{-32}
o-Xylene	1.42×10^{-60}	1.70×10^{-34}	3.77×10^{-1}	6.66×10^{-86}
2-Ethylhexanol	2.95×10^{-13}	8.92×10^{-7}	1.48×10^{-4}	1.45×10^{-11}

Supplementary Table S6. Correlation and regression analysis between profiled metabolites.

Metabolite		Spearman		Kendall		Pearson		Regression	Corrected Regression
**#1**	**#2**	**ρ**	**p**	**τ**	**p**	**r**	**p**	**p**	**p**
Toluene	*p*-Xylene	0.907	3.34×10^{-27}	0.733	3.01×10^{-19}	0.748	9.91×10^{-14}	9.91×10^{-14}	9.91×10^{-13}
Toluene	*m*-Xylene	0.846	3.30×10^{-20}	0.653	1.30×10^{-15}	0.784	1.03×10^{-15}	1.03×10^{-15}	1.03×10^{-14}
Toluene	*o*-Xylene	0.827	1.08×10^{-18}	0.631	1.07×10^{-14}	0.807	3.27×10^{-17}	3.27×10^{-17}	3.27×10^{-16}
Toluene	2-Ethylhexanol	-0.585	1.02×10^{-7}	-0.296	2.89×10^{-4}	-0.577	1.72×10^{-7}	1.72×10^{-7}	1.72×10^{-6}
p-Xylene	*m*-Xylene	0.952	1.42×10^{-36}	0.828	3.89×10^{-24}	0.879	1.44×10^{-24}	1.44×10^{-23}	1.44×10^{-22}
p-Xylene	*o*-Xylene	0.932	1.03×10^{-31}	0.795	2.28×10^{-22}	0.894	2.05×10^{-25}	2.05×10^{-25}	2.05×10^{-24}
p-Xylene	2-Ethylhexanol	-0.64	2.48×10^{-9}	-0.383	2.74×10^{-6}	-0.425	2.46×10^{-4}	2.46×10^{-4}	2.46×10^{-3}
m-Xylene	*o*-Xylene	0.979	8.45×10^{-49}	0.892	8.74×10^{-28}	0.995	3.22×10^{-70}	3.22×10^{-70}	3.22×10^{-69}
m-Xylene	2-Ethylhexanol	-0.679	1.03×10^{-10}	-0.424	2.03×10^{-7}	-0.512	6.03×10^{-6}	6.03×10^{-6}	6.03×10^{-5}
o-Xylene	2-Ethylhexanol	-0.697	2.14×10^{-11}	-0.441	6.69×10^{-8}	-0.523	3.38×10^{-6}	3.38×10^{-6}	3.38×10^{-5}

Supplementary Table S7. Headspaced GC-MS instrumentation condition for targeted metabolite profling.

**GC (Trace 1310) front inlet**	
split/splitless mode	splitless
Carrier mode	Constant flow
Inlet temperature	270 ℃
Split flow	20 mL/min
Splitless time	3 min
Carrier flow	1 mL/min
Purge flow	5 mL/min
**Headspace (TriPlus 500)**	
Injecting method	GC headspace
Syringe volume	2.5 mL
Sample draw	0.8 mL
Agitator temperature	90 ℃
Incubation time	15 min
**MS (ISQ 7000)**	
MS transfer line temperature	280 ℃
Ion source temperature	280 ℃
Ionization mode	EI
Scan time	1 min
Mass range	36 – 450 m/z
Scan time	0.4 sec

References

1. Forny, P., et al., *Integrated multi-omics reveals anaplerotic rewiring in methylmalonyl-CoA mutase deficiency.* Nature metabolism, 2023. **5**(1): p. 80–95.
2. Luciani, A., et al., *Impaired mitophagy links mitochondrial disease to epithelial stress in methylmalonyl-CoA mutase deficiency.* Nature communications, 2020. **11**(1): p. 970.
3. Liao, Y., et al., *Amino acid is a major carbon source for hepatic lipogenesis.* Cell Metabolism, 2024. **36**(11): p. 2437–2448. e8.
4. Deja, S., et al., *Hepatic malonyl-CoA synthesis restrains gluconeogenesis by suppressing fat oxidation, pyruvate carboxylation, and amino acid availability.* Cell metabolism, 2024. **36**(5): p. 1088–1104. e12.
5. Jin, S., et al., *ALDH2 (E487K) mutation increases protein turnover and promotes murine hepatocarcinogenesis.* Proceedings of the National Academy of Sciences, 2015. **112**(29): p. 9088–9093.
6. Chang, Y.-C., et al., *A common East-Asian ALDH2 mutation causes metabolic disorders and the therapeutic effect of ALDH2 activators.* Nature communications, 2023. **14**(1): p. 5971.
7. Consortium, G., *The GTEx Consortium atlas of genetic regulatory effects across human tissues.* Science, 2020. **369**(6509): p. 1318–1330.
8. Laboratory, D.A., et al., *Genetic effects on gene expression across human tissues.* Nature, 2017. **550**(7675): p. 204–213.
9. Digre, A. and C. Lindskog, *The Human Protein Atlas—Spatial localization of the human proteome in health and disease.* Protein science, 2021. **30**(1): p. 218–233.
10. Donovan, M.K., et al., *Cellular deconvolution of GTEx tissues powers discovery of disease and cell-type associated regulatory variants.* Nature communications, 2020. **11**(1): p. 955.
11. Niu, L., et al., *Dynamic human liver proteome atlas reveals functional insights into disease pathways.* Molecular Systems Biology, 2022. **18**(5): p. e10947.
12. Fromenty, B. and M. Roden, *Mitochondrial alterations in fatty liver diseases.* Journal of hepatology, 2023. **78**(2): p. 415–429.
13. Park, S. and M.N. Hall, *Metabolic reprogramming in hepatocellular carcinoma: mechanisms and therapeutic implications.* Experimental & Molecular Medicine, 2025: p. 1–9.
14. Chen, C.-H., et al., *Targeting aldehyde dehydrogenase 2: new therapeutic opportunities.*

Physiological reviews, 2014. **94**(1): p. 1–34.

15. Park, K.C., et al., *Disrupted propionate metabolism evokes transcriptional changes in the heart by increasing histone acetylation and propionylation.* Nature cardiovascular research, 2023. **2**(12): p. 1221–1245.
16. Reichardt, N., et al., *Phylogenetic distribution of three pathways for propionate production within the human gut microbiota.* The ISME journal, 2014. **8**(6): p. 1323–1335.
17. Cani, P.D., et al., *Microbial regulation of organismal energy homeostasis.* Nature metabolism, 2019. **1**(1): p. 34–46.
18. Fan, Y. and O. Pedersen, *Gut microbiota in human metabolic health and disease.* Nature Reviews Microbiology, 2021. **19**(1): p. 55–71.
